# Endoscopic Coagulation with Clipping for Colonic Diverticular Bleeding: A Practical Hemostatic Strategy

**DOI:** 10.1055/a-2889-0264

**Published:** 2026-06-24

**Authors:** Toru Matsui, Chihiro Tanaka, Atsushi Horie, Yusuke Niwa, Masatoshi Satoh, Oki Nakano, Hiromitsu Oka, Yutaka Honda, Masaaki Takamura

**Affiliations:** 1 Department of Gastroenterology 37012 Nagaoka Chuo General HospitalNiigataJapan

**Keywords:** endoscopy lower GI tract, lower GI bleeding, endoscopy upper GI tract, non-variceal bleeding, diagnosis and imaging (inc chromoendoscopy, NBI, iSCAN, FICE, CLE)

## Abstract

**Background and study aims** Endoscopic management of colonic diverticular bleeding remains challenging. Endoscopic coagulation with clipping (ECC) has been performed at our institution, but real-world outcome data remain limited. We aimed to describe the clinical outcomes and safety of ECC.

**Patients and methods** We conducted a single-center retrospective case series of consecutive patients with colonic diverticular bleeding who underwent ECC for endoscopically confirmed diverticular bleeding between January 2017 and December 2025. Rebleeding was defined as clinically significant recurrent hematochezia within 30 days after initial hemostasis. Rebleeding rates and procedure-related adverse events were evaluated.

**Results** Among 256 patients presenting with hematochezia and clinically suspected diverticular bleeding, 238 underwent colonoscopy. Of these, 83 patients who underwent ECC for endoscopically confirmed diverticular bleeding were included in the analysis. Rebleeding within 30 days occurred in 7 patients (8.4%), including 6 cases within 7 days. Among the 83 patients treated with ECC, stigmata of recent hemorrhage were identified in 81 patients. Among these, rebleeding occurred in 3 of 54 patients (5.6%) with active bleeding and in 4 of 27 patients (14.8%) with nonbleeding visible vessels or adherent clots. Procedure-related adverse events were observed in 3 patients (3.6%), all managed conservatively.

**Conclusions** In this single-center case series, ECC was associated with a low rebleeding rate in this cohort. ECC may represent a practical hemostatic option when the bleeding diverticulum is clearly identified.

## Introduction

Colonic diverticular bleeding (CDB) is a common cause of acute lower gastrointestinal bleeding. Recent epidemiologic studies in developed countries have indicated that the prevalence of colonic diverticulosis exceeds 50% in individuals aged over 60 years. Among these patients, CDB accounts for approximately 20% of acute lower gastrointestinal bleeding cases requiring hospitalization. 1 2 3 4 These conditions pose a substantial clinical burden because of frequent rebleeding, with reported readmission rates ranging from 10% to 20%, 5 particularly among older patients with comorbidities. These factors highlight the importance of effective hemostatic strategies in reducing clinical and economic burdens.

Identifying the bleeding site in acute CDB remains challenging, with reported detection rates ranging from 17% to 50%, depending on the timing and diagnostic modalities. 6 7 8 9 Inadequate localization of the bleeding site may increase the risk of rebleeding and necessitate repeat procedures. Therefore, accurate identification of the bleeding site and effective hemostasis are crucial for preventing recurrence.

Key strategies to improve identification have been proposed, including adequate bowel preparation, contrast-enhanced computed tomography (CT) before endoscopy, 10 and emergency endoscopy within 24 h of onset. 7 9 11 12

Once the source is identified, reliable hemostasis is required. Conventional endoscopic clipping is widely used; however, recent studies have reported favorable outcomes with endoscopic band ligation (EBL). Reported rebleeding rates range from 9% to 15% for EBL compared with 15–22% for clipping. 3 13 14 15 However, EBL requires reinsertion of the endoscope and specialized equipment, which may pose practical challenges in emergency settings. Other techniques, including over-the-scope clips (OTSCs) and hemostatic powders like PuraStat, have been introduced but are limited by cost and uncertain efficacy. 16 17

Endoscopic coagulation with clipping (ECC) is a technique involving thermal coagulation of the bleeding vessel followed by clip reinforcement, and it has been applied at our center as a practical and effective method for diverticular bleeding. However, real-world clinical data specifically evaluating ECC as a primary hemostatic strategy remain limited. Although thermal coagulation is widely used for hemostasis in upper gastrointestinal bleeding, coagulation hemostasis has not been widely adopted for CDB worldwide. In centers experienced in colorectal endoscopic submucosal dissection (ESD), thermal coagulation is commonly used for intraoperative hemostasis. For large lesions, prophylactic clip closure of the resection site is also performed to prevent delayed bleeding and perforation. 18 19 This rationale also underlies ECC, in which postcoagulation clipping may reduce the risk of perforation and rebleeding. Nevertheless, coagulation hemostasis is generally avoided in CDB because the thin wall of the pseudodiverticulum increases the risk of perforation, and the safety of this approach remains under-investigated, with only a few reports evaluating adverse events. We therefore aimed to describe the clinical outcomes of ECC in patients with CDB.

## Methods

### Patients and Study Design

**Fig. 1:**
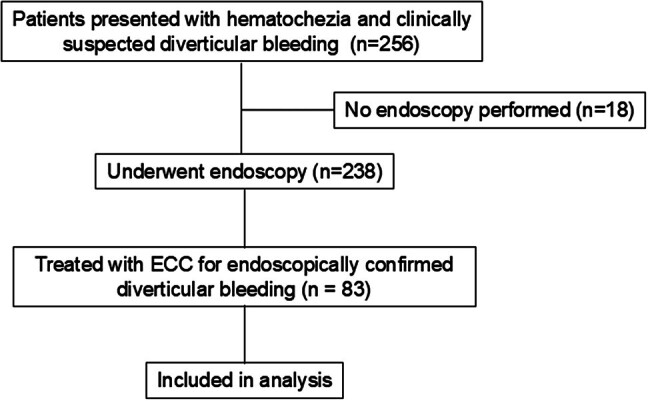
Flowchart of patient selection. Of the 256 patients with suspected colonic diverticular bleeding, 238 underwent colonoscopy during the study period. Among these, 83 patients were treated with ECC and were included in the present analysis. ECC: endoscopic coagulation with clipping; CDB: colonic diverticular bleeding.

This retrospective single-center case series included consecutive patients presenting with hematochezia and clinically suspected CDB at our institution between January 1, 2017, and December 31, 2025. Patients who did not undergo colonoscopy were excluded from the final analysis. Diverticular bleeding was diagnosed based on endoscopic findings and categorized as definitive or presumptive according to previously published criteria. ECC was performed when active bleeding or stigmata of recent hemorrhage (SRH) from a diverticulum was identified during the index or subsequent colonoscopy. The study flow is summarized in Fig. 1 .

### Inclusion and Exclusion Criteria

Patients were eligible if they were admitted with hematochezia and clinically suspected CDB between January 2017 and December 2025 and underwent colonoscopy at our institution.

Exclusion criteria were (1) upper gastrointestinal bleeding; (2) other colonic bleeding sources such as tumors, ischemic colitis, or hemorrhoids; and (3) incomplete clinical records or transfer before undergoing colonoscopy.

### Definition of Terms

Diverticular bleeding was classified as definitive or presumptive according to previously established criteria. 20 Definitive diverticular bleeding was defined as the presence of active bleeding or SRH—including active bleeding, a nonbleeding visible vessel, or an adherent clot—identified within a diverticulum during colonoscopy. Presumptive diverticular bleeding was defined as the absence of an alternative bleeding source after complete colonoscopic evaluation in a patient with colonic diverticulosis.

ECC was defined as thermal coagulation directly applied to the bleeding diverticulum, followed by clipping at the same site to reinforce hemostasis and reduce the risk of perforation. ECC was primarily indicated for cases with active bleeding or clearly identifiable SRH within a diverticulum. Hence, ECC may be less suitable when the bleeding source cannot be clearly identified or when adequate endoscopic visualization is not achieved. Careful consideration is required in patients with severe coagulopathy or unstable clinical condition. Appropriate precautions are necessary in patients with implanted cardiac devices. Rebleeding was defined as clinically significant recurrent hematochezia within 30 days after initial hemostasis. In this study, “endoscopy within 24 h” referred to colonoscopy performed within 24 h after the patient’s arrival at the hospital—not from the onset of bleeding. Adverse events were defined as perforation or clinically significant abdominal symptoms requiring additional management, assessed within 30 days postprocedure by review of medical records, imaging, and laboratory results.

### Endoscopic Procedures

**Fig. 2:**
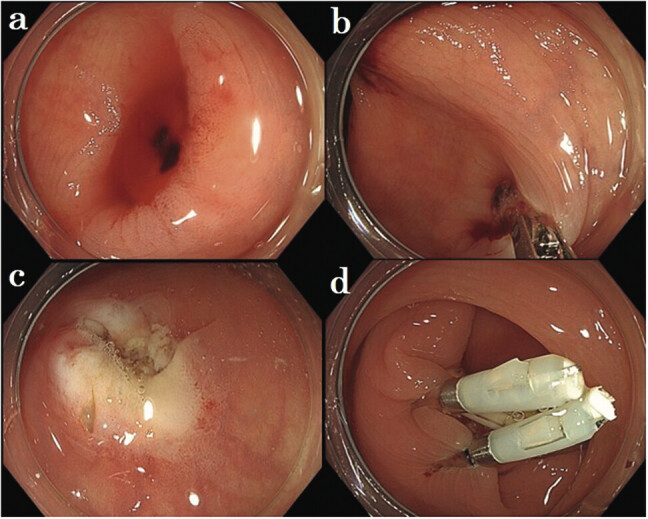
Hemostatic procedure using ECC. ( **a** ) A diverticulum identified as the bleeding source. ( **b** , **c** ) The bleeding site was grasped with monopolar hemostatic forceps, gently lifted, and coagulated. ( **d** ) The site was subsequently closed with clips to reinforce hemostasis and prevent perforation. ECC: endoscopic coagulation with clipping.

Hemostasis was performed using a Coagrasper (FD-411UR; Olympus, Tokyo, Japan) with a high-frequency generator (ICC 200, VIO 300D; Erbe, Tübingen, Germany), and soft coagulation mode (Effect 4, 60 W) was used in most cases. The bleeding point was grasped with the forceps, gently lifted, and coagulated for 2–3 s ( Fig. 2 ). After coagulation, 2–4 standard hemostatic clips were typically applied to the treated diverticulum. The clips were placed in a manner that approximated the edges of the diverticular orifice, effectively closing it in a purse-string-like fashion to reinforce hemostasis and minimize the risk of delayed perforation. Representative procedures were recorded and are available as Supplementary video 1.

### Statistical Analysis

Statistical analyses were performed using EZR (Saitama Medical Center, Jichi Medical University), a graphical user interface for R. Continuous variables are presented as median with interquartile range (IQR), and categorical variables as number and percentage.

Exploratory comparisons between patients with and without early rebleeding were conducted using the Mann–Whitney *U* test for continuous variables and Fisher’s exact test for categorical variables. These analyses were considered hypothesis-generating and interpreted cautiously due to the small number of rebleeding events ( *n* = 7). A two-sided *P* value < 0.05 was considered statistically significant.

### Ethics

This study was approved by the institutional review board of Nagaoka Chuo General Hospital (Approval No. 688). The study was conducted in accordance with the Declaration of Helsinki. Informed consent was obtained in the form of an opt-out on the hospital website.

## Results

Table 1 summarizes the baseline characteristics of the 83 patients treated with ECC. The median age was 75 years (IQR, 69–83), and 62 patients (74.7%) were male. Hypertension was present in 59 patients (71.1%), diabetes in 24 (28.9%), and chronic kidney disease (eGFR <60 mL/min/1.73 m ^2^ ) in 35 (42.2%). Antithrombotic agents were used in 34 patients (41.0%), and NSAIDs in 16 (19.3%). Contrast extravasation on CT was observed in 46 patients (55.4%), and colonoscopy within 24 hours of hospital arrival was performed in 74 patients (89.2%).

**Table 1:** Baseline characteristics of patients treated with ECC.

Variable	ECC cohort ( *n* = 83)
Age, years, median [IQR]	75.0 [69.0–83.0]
Male, *n* (%)	62 (74.7%)
BMI, kg/m ^2^ , median [IQR]	23.0 [21.4–25.5]
Hemoglobin before colonoscopy, g/dL, median [IQR]	10.0 [7.8–11.7]
Diabetes, *n* (%)	24 (28.9%)
Hypertension, *n* (%)	59 (71.1%)
Renal dysfunction (eGFR < 60), *n* (%)	35 (42.2%)
Ischemic heart disease, *n* (%)	11 (13.3%)
Cerebral infarction, *n* (%)	14 (16.9%)
NSAID use, *n* (%)	16 (19.3%)
Antithrombotic use, *n* (%)	34 (41.0%)
CT extravasation, *n* (%)	46 (55.4%)
Colonoscopy within 24 h, *n* (%)	74 (89.2%)

Abbreviations: ECC, endoscopic coagulation with clipping; IQR, interquartile range; BMI, body mass index; eGFR, estimated glomerular filtration rate; NSAID, non-steroidal anti-inflammatory drugs; CT, computed tomography. Comparisons are descriptive and not intended to imply causality.

Rebleeding within 30 days occurred in 7 patients (8.4%), including 6 cases within 7 days ( Table 2 ). Among patients in whom SRH were identified ( *n* = 81), rebleeding occurred in 3 of 54 patients (5.6%) with active bleeding and in 4 of 27 patients (14.8%) with nonbleeding visible vessels or adherent clots.

**Table 2:** Clinical outcomes in the ECC group.

Clinical outcome	ECC group ( *n* = 83)	Remarks
Rebleeding within 30 days	7/83 (8.4%)	One case occurred 12 days after initial treatment
Rebleeding within 7 days	6/83 (7.2%)	
**Interventions at rebleeding**		
ECC retreatment	3 cases	
Endoscopic clipping	1 case	
TAE followed by surgery	1 case	Endoscopic intervention was technically challenging due to poor scope maneuverability
Observation only	2 cases	
Adverse events	3 cases (3.6%)	Two cases of abdominal pain and one case of delayed perforation; all resolved with conservative management

Abbreviations: ECC, endoscopic coagulation with clipping; TAE, transcatheter arterial embolization.

Management of rebleeding included repeat ECC in 3 patients, endoscopic clipping in 1, transcatheter arterial embolization followed by surgery in 1, and observation alone in 2 patients.

Procedure-related adverse events were observed in 3 patients (3.6%), including abdominal pain in two patients and delayed perforation in one patient. All adverse events were managed conservatively without the need for surgical intervention.

Exploratory univariate analysis within the ECC cohort did not identify any clear differences between patients with and without early rebleeding ( Table 3 ). Rebleeding tended to be more frequent in patients with nonbleeding visible vessels or adherent clots than in those with active bleeding, although this difference did not reach statistical significance ( Table 4 ).

**Table 3:** Comparison of clinical characteristics between rebleeding and non-rebleeding groups in the ECC cohort. Variables selected based on clinical relevance to bleeding severity, treatment decision-making, or rebleeding risk.

Clinical variable	Rebleeding group ( *n* = 7)	Non-rebleeding group ( *n* = 76)	*P* value
Age (years), median [IQR]	72.0 [68.5–81.0]	75.0 [69.0–83.0]	0.87
BMI (kg/m ^2^ ), median [IQR]	21.9 [20.2–24.7]	23.2 [21.6–25.5]	0.441
Hemoglobin (g/dL), median [IQR]	8.8 [8.3–9.4]	10.1 [7.7–11.8]	0.427
Antithrombotic drug use, *n* /N (%)	3/7 (42.9%)	31/76 (40.8%)	1.000
CT extravasation, *n* /N (%)	2/7 (28.6%)	44/76 (57.9%)	0.233
SRH classification (AB/NBVV/AC)	3 / 0 / 4	51 / 5 / 18	0.189
Blood transfusion, *n* /N (%)	3/7 (42.9%)	21/76 (27.6%)	0.407
Endoscopy within 24 h, n/N (%)	6/7 (85.7%)	68/76 (89.5%)	0.567

Abbreviations: ECC, endoscopic coagulation with clipping; IQR, interquartile range; SRH, stigmata of recent hemorrhage; AB, active bleeding; NBVV, non-bleeding visible vessel; AC, adherent clot.

**Table 4:** Comparison of the rebleeding rates and CT extravasation between patients with AB and NBVV/AC-type SRH.

SRH type	Total (n)	Non-rebleeding, *n* (%)	Rebleeding, *n* (%)	CT extravasation, *n* (%)	*P* value (rebleeding)	*P* value (CT extravasation)
AB	54	51 (94.4%)	3 (5.6%)	34 (63.0%)	–	–
NBVV or AC	27	23 (85.2%)	4 (14.8%)	10 (37.0%)	0.214	0.035

## Discussion

**Video 1** Endoscopic coagulation with clipping (ECC) for colonic diverticular bleeding. Active bleeding from a colonic diverticulum is treated using Coagrasper soft coagulation, followed by clipping to reinforce hemostasis.


In the present study, we evaluated the clinical outcomes of ECC for colonic diverticular bleeding. ECC was associated with a 30-day rebleeding rate of 8.4% and no serious treatment-related adverse events. These outcomes may reflect our treatment strategy, as endoscopic intervention was generally performed only when active bleeding or clear SRH was identified. In our cohort, ECC was performed predominantly in patients with SRH (81/83), while in the remaining two patients without clearly identifiable SRH, recent diverticular bleeding was suspected during endoscopy and supported by contrast extravasation on CT. This reflects a common real-world clinical scenario in which definitive endoscopic therapy is attempted only when the bleeding source can be clearly identified. In particular, intervention is recommended when SRH—such as AB, NBVV, or AC—are present. 21 22 23 Accordingly, ECC was mainly performed when the bleeding point was clearly identified and endoscopically accessible, reflecting real-world clinical practice. This selection inevitably introduces bias, but it also means that ECC was applied in scenarios where hemostasis is most likely to be technically feasible and effective.

Although endoscopic clipping was traditionally the most common technique for CDB, its effectiveness has been increasingly questioned, particularly in cases where indirect clipping is performed without clear visualization of the bleeding source, which has been associated with relatively high rebleeding rates. 24 However, the present study was not designed to compare ECC with clipping or EBL, and the results should therefore be interpreted as descriptive outcomes within an ECC-treated cohort. Recent studies have suggested that direct clipping, in which the bleeding vessel is directly targeted, results in significantly lower rebleeding rates than indirect clipping. These findings from previous studies indicate that the success of endoscopic hemostasis depends not only on the technique used but also on the visibility of the bleeding source.

In our cohort, patients with AB-type SRH, representing active bleeding, had a markedly lower rebleeding rate than those with NBVV- or AC-type SRH (5.6% vs. 14.8%). This difference likely reflects the greater visibility and accessibility of the bleeding point in AB-type cases, facilitating more accurate and effective hemostasis using ECC. Although this difference did not reach statistical significance, the trend warrants confirmation in larger, multicenter studies. In contrast, NBVV- or AC-type lesions may involve less clearly defined targets, making durable hemostasis more difficult to achieve. These results underscore the importance of SRH type in guiding both treatment decisions and prognostic expectations for diverticular bleeding, and also highlight the challenges in comparing outcomes between different hemostatic techniques, particularly in retrospective analyses.

Since visibility of the bleeding point is critical for successful hemostasis, EBL has been increasingly adopted in patients with clearly visualized bleeding diverticula. 13 14 25 26 Although EBL has demonstrated favorable outcomes in selected cases, the procedure requires withdrawal and reinsertion of the endoscope after mounting the ligation device, which may be challenging in emergency settings. These technical considerations may influence treatment selection in real-world practice.

In contrast, ECC can be performed immediately using standard hemostatic forceps and clips without scope exchange, which may be advantageous in emergency settings.

Coagulation therapy for diverticular bleeding is generally avoided because diverticula form at sites where the vasa recta penetrate the muscularis propria, creating natural weak points in the colonic wall. 27 Thermal injury to these thin-walled structures may increase the risk of adverse events, including delayed perforation, particularly if excessive coagulation is applied. 28 29 Nevertheless, when performed under controlled conditions, several factors may contribute to its relative safety. Specifically, using low-power soft coagulation to limit thermal penetration and applying clips to reinforce the treated area provide a double safeguard against transmural injury. A similar concept is applied in colorectal endoscopic submucosal dissection (ESD), where prophylactic clip closure is often performed after coagulation of exposed vessels to reduce the risk of delayed bleeding or perforation. In our study, only one case (1.2%) of delayed perforation occurred, which resolved with conservative management, and no immediate perforation or fatal adverse events were observed. These findings suggest that ECC can be performed safely when coagulation is applied carefully. However, given the inherent risk of thermal injury associated with coagulation, ECC should not be considered a standard first-line treatment but rather a complementary option when the bleeding point is clearly identified.

Importantly, the observed 30-day rebleeding rate of 8.4% falls within the range reported for endoscopic hemostasis in previous studies. 13 14 ECC is technically straightforward and cost-effective, without the need for specialized devices—unlike hemostatic powders (e.g., PuraStat) or OTSC systems, which may require additional equipment and cost. 30 31 Given its technical simplicity and the observed safety profile in this study, ECC may represent a practical treatment option.

Our study has some limitations. First, this was a retrospective, single-center study without a direct comparator group. As ECC has been our primary hemostatic strategy for definitive CDB, establishing a balanced control group was not feasible. Additionally, the observation period was limited to 30 days; thus, long-term outcomes remain unknown.

Second, most ECC-treated diverticular bleeding cases occurred in the right colon (69%), reflecting the general distribution pattern observed in Asian populations. As left-sided bleeding is more common in Western countries, the applicability to those settings may be limited, although ECC was also applied to left-sided cases in this study with similar short-term outcomes. In addition, we did not perform a detailed analysis according to the location of the bleeding diverticulum, as the number of rebleeding events was limited.

Third, there is a potential selection bias, as ECC and EBL are generally performed only in patients with clearly identified SRH, whereas clipping is often used even when SRH is absent. This may partly account for differences in reported rebleeding rates among techniques. In addition, detailed classification of the bleeding site within the diverticulum (e.g., dome vs. neck) was not consistently available; therefore, location-specific differences in adverse events could not be evaluated. Furthermore, multivariate analysis was not feasible due to the limited number of rebleeding cases. Therefore, these findings should be interpreted as descriptive observations within a selected cohort rather than evidence of comparative effectiveness. Further multicenter studies are warranted to assess its applicability in broader clinical settings. ECC may serve as a practical alternative hemostatic strategy in centers where EBL is not readily available or technically difficult to perform.

In conclusion, ECC was associated with a low rebleeding rate in this cohort. ECC may be a feasible and practical hemostatic option in selected patients when the bleeding source is clearly identified.
